# Patient-Reported Outcomes following Genetic Testing for Familial Hypercholesterolemia, Breast and Ovarian Cancer Syndrome, and Lynch Syndrome: A Systematic Review

**DOI:** 10.3390/jpm11090850

**Published:** 2021-08-27

**Authors:** Rachele M. Hendricks-Sturrup, Lucson Joseph, Christine Y. Lu

**Affiliations:** 1 Department of Population Medicine, Harvard Pilgrim Health Care Institute and Harvard Medical School, Boston, MA 02215, USA; christine_lu@hphci.harvard.edu; 2 Duke-Margolis Center for Health Policy, Duke University, Washington, DC 20004, USA; 3 Tanner Health System, Carrollton, GA 30117, USA; lucjoseph15@gmail.com

**Keywords:** patient-reported outcomes, genetic testing, familial hypercholesterolemia, breast and ovarian cancer syndrome, Lynch syndrome, genomics, germline testing

## Abstract

Background: Patient-reported outcomes (PROs) and PRO measures (PROMs) are real-world evidence that can help capture patient experiences and perspectives regarding a clinical intervention such as genetic testing. Objective: To identify and capture methods and qualitative PRO themes among studies reporting PROs following genetic testing for FH, breast and ovarian cancer syndrome, and Lynch syndrome. Methods: A systematic review was conducted via PubMed/MEDLINE, EMBASE, and Yale University’s TRIP Medical Databases on articles published by April 2021. Results: We identified 24 studies published between 1996 and 2021 representing 4279 participants that reported PROs following genetic testing for FH, breast and ovarian cancer syndrome, and Lynch syndrome. Studies collected and reported PROs from validated PROM instruments (*n* = 12; 50%), validated surveys (*n* = 7; 26%), and interviews (*n* = 10; 42%). PRO themes ranged across all collection methods (e.g., psychological, knowledge, coping and satisfaction, concern about stigma/discrimination, etc.). Conclusions: Important gaps identified include (1) most studies (*n* = 18; 75%) reported PROs following genetic testing for breast and ovarian cancer, and (2) populations reporting PROs overall were largely of White/Caucasian/Northern European/Anglo-Saxon descent. We offer recommendations and describe real-world implications for the field moving forward.

## 1. Introduction

Patient-reported outcomes (PROs) are surrogate endpoints that can help clinicians and researchers understand and document the patient journey [1]. PROs most notably capture data about personal utility from the patient perspective and includes patient preferences, complaints, and/or opinions before and following an intervention. When used in conjunction with more novel forms of person-generated health data, PROs offer valuable insights into the daily experiences of managing disease burden and real-world effectiveness and utility of interventions from the patient point of view [2,3].

For instance, PROs provide insights into humanistic (e.g., emotional status) and economic (e.g., out-of-pocket costs) outcomes following an intervention, which typically include anxiety or depression levels, functional status, and overall experience and satisfaction [4]. PROs are, therefore, valuable because they provide insights into what or how patients feel as well as how they value clinical interventions [2].

Generally, five broad categories of PROs have been described in the literature and can be quantitatively assessed as PRO measures (PROMs) using surveys: health-related quality of life, functional status, symptoms and symptom burden, health behaviors, and the patient’s health care experience [5]. Each PRO category is accompanied by its own strengths and limitations when PROs are collected and processed in a structured form as PROMs (see Table 1, adopted by Cella et al., as examples of PROs) [5].

### Background and Purpose

PROs have provided meaningful insight into individuals’ and their family members’ real or perceived genetic health status and offer opportunities for patients to convey their concerns, including experiences with insurance discrimination and/or access issues with insurance coverage for genetic testing and subsequent treatments [6,7]. Cella et al.’s definition of PROs in Table 1 only partially fits within the ‘personal utility’ concept of genetic testing that is emerging in the literature [5,6]. Notably, a systematic literature review of empirical studies conducted by Kohler et al. delineated the personal utility of genetic testing, capturing 15 distinct elements of personal utility within two domains: personal outcomes (affective, cognitive, and behavioral) and social outcomes [6]. Therefore, further exploration, crosswalk analysis, and interpretation of PROs following genetic testing are warranted for a variety of medical conditions to inform both clinical practice and policy [6,8,9].

Additionally, prior work has summarized studies on how PROs from clinical genetic testing can translate into patient empowerment [10]. In our prior work, we observed and acknowledged that PROs were underreported following genetic testing for familial hypercholesterolemia (FH) [11]. We also concluded that further investigations are needed to examine PROs following genetic testing for familial conditions falling under the United States (US) Centers for Disease Control and Prevention (CDC) Tier 1 genomic application category (FH, hereditary breast and ovarian cancer syndrome, and Lynch syndrome) [11]. Murray et al. described in their National Academy of Medicine discussion report ‘Tier 1’ as ‘those genes with high penetrance (the probability that disease will appear when a disease-related genotype is present), well-understood links to disease, and well-established, effective interventions that result in substantial prevention or mitigation of disease or disease risk’ [12].

Therefore, the purpose of this review is two-fold with regard to PROs and PROMs following genetic testing for FH, breast and ovarian cancer syndrome, and Lynch syndrome: (1) examine overarching qualitative themes among PROs and PROMs collected, measured, and reported in the literature; and (2) identify opportunities to improve existing PRO/PROM categories, collection methods, and measures to more accurately and appropriately capture and describe patient needs, perspectives, and personal utility.

To understand the larger role and impact of PROs following genetic testing for these conditions, our systematic literature review focused on the following PICO [13]:

P: Patients and/or consumers with breast and ovarian cancer syndrome, Lynch syndrome, and familial hypercholesterolemia.

I: Genetic testing (clinical or direct-to-consumer with a clinical intermediary) for molecular diagnosis/confirmation of breast and ovarian cancer syndrome, Lynch syndrome, and FH.

C: Patients and/or consumers with breast and ovarian cancer syndrome, Lynch syndrome, and familial hypercholesterolemia who did not undergo genetic testing.

O: PROs, personal financial/insurance coverage outcomes, humanistic outcomes (privacy, discrimination, family planning, education), and personal behavioral (treatment adherence, treatment change).

## 2. Materials and Methods

### 2.1. Background and Purpose

Our systematic review was divided into the following four steps in order to establish rigor in the process: (a) Database Identification and Selection, (b) Selection of Studies, (c) Data Extraction and Synthesis, and (d) Data Analysis. Preferred Reporting Items for Systematic Reviews (PRISMA) checklist items and guidelines were followed to the extent possible (www.prisma-statement.org; accessed 28 July 2020), as our review entailed a meta-synthesis of qualitative themes or data extracted from selected studies that met our inclusion criteria. Our meta-synthesis strategy draws on previous methods used by Seyedfatemi et al. in which qualitatively themes were identified and assessed in a manner that might inspire the development of a more comprehensive quantitative survey or analysis based on identified and subsequently defined themes [14].

### 2.2. Database Identification and Selection

Search queries for original research reports were run in PubMed/MEDLINE, EMBASE, and Yale University’s TRIP Medical Database in April 2021 by one author (R.M.H-S.) using Boolean strings with both MeSH and general terms. When available, filters were used to identify human studies as well as systematic literature reviews that captured human studies focused on our PICO. Specifically, our search strategy (available from the authors on request) was adapted to each database, using terms focused on PROs and/or PROMs: PRO-related terms (e.g., anxiety, genetic testing, function, satisfaction, experience, education, psychology, privacy, knowledge, attitude, insurance coverage, healthcare expenditures/cost, cost-sharing, treatment/surveillance adherence); genetic testing (clinical or direct-to-consumer with a clinical intermediary); and breast and ovarian cancer syndrome, Lynch syndrome, and/or FH. The reference lists of relevant systematic literature reviews were also examined.

### 2.3. Selection of Studies

Articles published by or before April 2021 were reviewed for relevance to our stated PICO. The authors carefully selected and reviewed studies reporting PROs following genetic testing for molecular diagnosis/confirmation of breast and ovarian cancer syndrome, Lynch syndrome, and/or FH. Original research articles were excluded if they were not published in the English language and if they were case reports (*n* = 1). Titles and abstracts were independently screened and jointly reviewed by two authors (R.H-S. and L.J.) for relevance and the inclusion criteria (published in English; human study; original research reports focused on PROs following genetic testing for breast and ovarian cancer syndrome, Lynch syndrome, and/or FH). Studies focused on PROs following germline preimplantation genetic diagnosis and prenatal diagnosis for breast and ovarian cancer syndrome, Lynch syndrome, and/or FH were excluded. Selected papers were downloaded and merged into Zotero software (www.zotero.org; accessed 16 July 2021) for reference purposes, and duplicates were removed.

### 2.4. Data Extraction and Synthesis

Full-text articles that were selected for inclusion were reviewed to identify key qualitative themes regarding PROs following genetic testing for breast and ovarian cancer syndrome, Lynch syndrome, and/or FH. Attention was also paid to the geographic location of each study and whether the studies were qualitative, quantitative, or mixed-methods.

Two authors (R.H-S. and L.J.) performed data extraction using a Microsoft Excel form. One author (R.H-S.) developed and piloted this form on five studies before a second reviewer (L.J.) performed a second review to ensure completeness. A third senior author (C.Y.L.) performed a third review of the form to ensure accuracy, clarity, and relevance.

The following data were extracted from each study:

- Author/year;
- Aims and purpose of the study;
- Number of participants tested;
- Number of patients surveyed or interviewed following genetic testing;
- Adult and/or pediatric population;
- Age range of participants;
- Gender (% females tested);
- Geographic region/location;
- Reporting of race or ethnicity;
- Disease;
- Genes or alleles examined;
- PRO/PROM instrument used (i.e., study design and data collection methods);
- PRO themes reported.

Two authors (R.H-S. and L.J.) extracted the data on all studies meeting the inclusion criteria with ongoing discussion to ensure consistency, followed by a final discussion with a third senior author (C.Y.L.) to ensure accuracy and clarity regarding both descriptive information and qualitative themes extracted per article and overall.

### 2.5. Assessment of Methodological Quality

Although qualitative, quantitative, and mixed-methods studies were included in our analysis, we drew on previous methods used that involved the application of the Critical Appraisal Skills Programme (CASP) tool checklist for qualitative studies and trials to assess the quality of studies selected for inclusion that captured key qualitative themes along the stated PICO (www.casp-uk.net; accessed 28 July 2020) [15]. For instance, although some of the studies selected for inclusion quantitatively assessed study participant outcomes within specific qualitative domains via a survey (e.g., anxiety, depression, distress, etc.), we aimed to only capture those specific qualitative domains within each survey tool as well as the name/type of survey instruments or tools used.

## 3. Results

**Figure 1:**
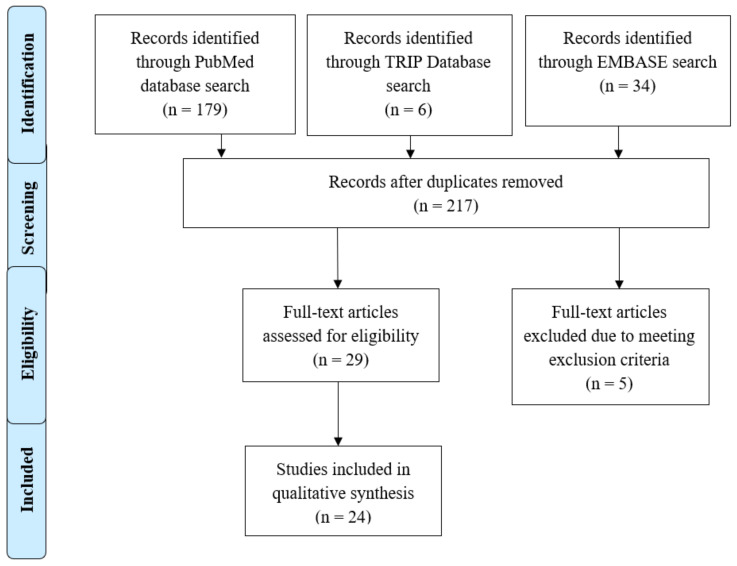
PRISMA Flow Diagram.

The search strategy identified 218 articles; titles and abstracts were screened for relevance to the stated PICO (see Figure 1). A total of 216 records were screened by title and abstract by two authors (R.H-S. and L.J.) after removing duplicates. The two authors reached an overall agreement rate of >95%. After reviewing titles and abstracts for relevance to the stated PICO, 29 papers were retrieved for full-text screening by three authors (R.H-S., C.Y.L., and L.J.). A total of 24 articles met all inclusion and exclusion criteria after full-text review. Of these, four articles that met inclusion criteria were identified and extracted from a scoping global literature review (Jones et al., 2018, and Pang et al., 2018, extracted from Hendricks-Sturrup et al., 2020) and a systematic literature review (Dudok deWit et al., 1998, and Croyle et al., 1997, extracted from Broadstock et al., 2000) [11,16,17,18,19,20].

### 3.1. Article Characteristics

Table 2 lists the titles, authors, and countries for each of the 24 included studies [16,17,18,20,21,22,23,24,25,26,27,28,29,30,31,32,33,34,35,36,37,38,39]. Table 1 also includes, for each study, the genetic disease focus or foci of each study (regarding PROs following genetic testing for breast and ovarian cancer syndrome, Lynch syndrome, and/or FH) and whether the study was qualitative, quantitative, or mixed-methods in nature. Studies were published between 1996 and 2021. Most studies were conducted in the United States (USA; n = 14), with one of these 14 studies conducted jointly between researchers within the USA and Canada. Most studies (n = 18) focused on PROs following genetic testing for breast and ovarian cancer, with one of these 18 studies focusing on breast, ovarian, and endometrial/uterine cancer. Most studies were quantitative (n = 11), followed by qualitative (n = 7), and mixed-methods studies (n = 4). Two (n = 2) studies did not explicitly report the study methods used to arrive at the qualitative themes captured.

### 3.2. Study and Participant Population Characteristics

There was a total of 5654 participants tested across all studies included in the review. One study (Palmquist et al., 2010) did not report the number of participants tested [35]. A total of 4279 participants across all studies provided PROs following genetic testing. Participants were reported as genetically tested for BRCA1/2 (breast and ovarian cancer), MLH1, MSH2, MSH6, PMS2, or EPCAM (Lynch syndrome), and LDLR, APOB, or PCSK9 (FH). deWit et al. and Schneider et al. did not report specific variants tested, although the disease focus of the study was breast and ovarian cancer and Lynch syndrome, respectively [18,36]. Voorwinden et al. reported testing for BRCA1/2 for their breast and ovarian cancer participant population but did not specify variants tested for their Lynch syndrome participant population [38].

Two studies collected PROs at specified time intervals (Meiser et al., 2018, at 1 year following testing; and Manchanda et al., 2019, at baseline, 1 year, 2 years, and 3 years following testing, although the number of participants reporting PROs at 1 year (n = 796) was included in the total number of participants who provided PROs following genetic testing) [32,33]. One study collected PROs from 192 participants, with 140 participants completed qualitative interviews (Lerman et al., 1996) [27]. Two studies (Pang et al., 2018, and Lee et al., 2002) did not state the number of participants reporting PROs following genetic testing [17,26]. The number of participants surveyed or interviewed across all studies ranged from 7 to 984.

Participants comprised of pediatric and adult populations. Adult ages across the 24 studies ranged from 18 to 91 years. Two studies did not explicitly report participants’ ages (Luba et al., 2018, and Manchanda et al., 2019) [29,32]. Studies ranged from 42% to 100% female, although most of the studies were majority or at least 50% female (n = 23; notwithstanding Pang et al., 2018, who did not report the genders or gender identities of the pediatric population surveyed) [17]. Fifteen studies (n = 15) reported participant populations’ races/ethnicities; nine studies (n = 10) did not explicitly report the races/ethnicities of their study participants [7,17,18,24,28,30,34,36,38]. All 15 of these studies reported majority participants of White/Caucasian/Northern European/Anglo-Saxon descent (78–100%).

### 3.3. PROs from Validated PROM Instruments

Several studies collected and reported PROs from validated PROM instruments (n = 12) following genetic testing (see Table 3). Of these, 11 studies reported PROs following genetic testing for breast and ovarian cancer syndrome (including breast, ovarian, and endometrial/uterine cancer). Only two studies (Esplen et al. and Voorwinden et al.) used validated PROMs to evaluate PROs following genetic testing for Lynch syndrome and none for FH genetic testing [23,38].

The State-Trait Anxiety Inventory instrument was used by Beri et al., Croyle et al., and Esplen et al. to measure participants’ state of anxiety following genetic testing and return of results [20,21,23]. Manchanda et al. used the Health Anxiety Inventory to conduct this same or a similar assessment [32]. Beri et al., Manchanda et al., Meiser et al., and Mella et al. used the Hospital Anxiety and Depression Scale to measure general anxiety and/or depression levels among participants following testing and return of results [21,32,33,34]. Esplen et al. and Lerman et al. (1998) used the Center for Epidemiologic Studies for Depression Scale to measure the frequency and intensity of symptoms of depression following testing and return of results [23,28].

The Impact of Event Scale was used by Beri et al., Croyle et al., deWit et al., Esplen et al., Lerman et al. (1998; Intrusion Subscale of the Revised Impact of Event Scale), and Meiser et al. to measure participants’ levels of worry about developing the disease following genetic testing for the disease [18,20,21,23,28,33]. Voorwinden et al. used the Cancer Worry Scale to collect and measure participants’ worries about developing cancer, the test results’ influence on the participants’ mood, restrictions in daily activities, and worries about cancer occurrence in or among family members [38].

Meiser et al. used the Test-Related Distress and Positive Experiences instrument to collect and measure participants’ distress and positive experiences following testing [33]. Emotional Thermometers were used as an ad hoc instrument by Mella et al., who also used the Profile of Mood States instrument to capture the participants’ spectrum of emotional states following testing [34]. Voorwinden et al. used the General Health Questionnaire-12 to measure and assess participants’ emotional problems as an indicator of their psychological functioning [38]. Decision regret regarding surgical decision or choice and genetic testing choice was measured by Meiser et al. using the Decision Regret Scale [33].

The Cancer Genetics Knowledge scale instrument used by Beri et al. assessed participants’ knowledge about their genetic risk for cancer [21]. The Multidimensional Impact of Cancer Risk Assessment was used by Beri et al., Hughes Halbert et al., Manchanda et al., and Underhill-Blazey et al. to measure the impact of genetic test result disclosure and the study participants’ uncertainty about gene variants of unknown significance, clinical implications, best approaches for future screening and disease risk mitigation, and implications for developing and carrying de novo gene mutations associated with the disease [21,25,32,37]. Esplen et al. used the Perception of Lifetime Risk for Colorectal Cancer instrument to collect and measure the participants’ perceived lifetime risk of developing colorectal cancer following genetic testing and the Colonoscopy Screening instrument to determine if at-risk participants underwent colonoscopy screening within the past year [23]. Esplen also used the Demographic and Medical Information Questionnaire to assess participants’ personal history of cancer, demographic factors (e.g., ethnicity, age, sex, education, occupation, age at diagnosis, if applicable, etc.), genetic test result (i.e., positive or negative), and amount of time passed since the return of their genetic test results [23].

The Ways of Coping Questionnaire and Social Support Questionnaire were used by Esplen et al. to measure the participants’ coping styles (e.g., escape avoidance) and levels of social support (e.g., personal support from family and friends) following testing [23]. Esplen et al. also used the Quality of Life Index to measure the participants’ family functioning following testing or return of results [23]. Lerman et al. (1996) used the Medical Outcomes Study instrument to measure participants’ functional health status, specifically impairment in daily activities (i.e., role impairment) and sexual functioning following testing and return of results [27]. Although, Machanda et al. differed in that they used the Physical Health Component and Mental Health Component scale of the SF-12 instrument to measure the quality of life among participants [32].

### 3.4. PROs from Validated Surveys

Seven studies (n = 7) reported PROs from validated general surveys (see Table 4). The majority of these studies (n = 6) focused on breast and ovarian cancer (including breast, ovarian, and endometrial/uterine cancer). Two studies focused on Lynch syndrome, either alone or in tandem with breast and ovarian cancer. None of the studies used general surveys to evaluate PROs following FH genetic testing.

### 3.5. PROs from Qualitative Studies and Studies with Unreported PRO Data Collection Methods

Ten studies (n = 10) used qualitative data or other unstructured PRO data collected from study participants (see Table 5). Two studies (n = 2) reported PROs in the study discussion, but not the study results. Eight studies reported the use of qualitative interviews; one study reported the use of telephone interview assessments, and one study reported the use of focus group interviews. Two studies reported PROs but did not report data collection methods. Most of these studies (n = 8) were conducted among breast and ovarian cancer patients (including patients with endometrial/uterine cancer), although one of these studies also focused on patients with Lynch syndrome. Only two studies reported PROs following FH genetic testing.

### 3.6. Overall CASP Assessment

None of the studies that met the inclusion criteria were excluded following CASP appraisal, yet we observed some variation in their quality (see Table 6). Every study provided a clear statement of research aims, although only a third of the studies (n = 8) contained appropriate qualitative methodology largely because many of the studies reported qualitative PRO themes from quantitative PROM surveys. The majority of studies (n = 22) used a research design that was appropriate to address the research aims and research issue, with the exception of Jones et al. and Lee et al., whose research design was unclear or not clear enough to draw a distinct connection between the study aims and the methods [16,26]. Specifically, Jones et al. did not include details on qualitative data coding methods despite being mixed-methods studies [16]. Lee et al. did not report qualitative study methods, although they reported qualitative PRO results [26]. Every study used appropriate recruitment strategies.

One study (Croyle et al.) did not clearly describe the relationship between the researchers and study participants [20]. The authors did not acknowledge at the end of the report potential conflicts of interest, yet they noted a study limitation of sample bias and the likelihood of adverse psychological effects among individuals with varying levels of health knowledge and risk awareness. Every study reported rigorous data analysis, with the exception of Lee et al., whose study methods were not well-structured or unclear [26]. Despite these CASP assessment limitations, every study provided a clear statement of PRO findings, and most of the studies (n = 22) provided valuable knowledge around PROs following genetic testing for breast and ovarian cancer syndrome, Lynch syndrome, and/or FH.

## 4. Discussion

This systematic review examined two aspects of the existing literature on PROs following genetic testing for FH, breast and ovarian cancer syndrome, and Lynch syndrome: (1) qualitative themes within and among PROs and PROMs collected, measured, and reported to date; and (2) existing PRO/PROM collection methods and measures that, to date, have captured and described patient needs, perspectives, and personal utility.

Several PROM instruments were used across studies to measure and collect PROs along three overarching themes following genetic testing (see Table 3): (1) psychological, mood, emotional function or state (i.e., feelings of anxiety, depression, distress, regret, and worry); (2) knowledge and perceptions of their lifetime or genetic disease risk, uncertainty about managing their genetic disease risk, and health screening behaviors; and (3) coping style or mechanisms, social functioning and support, quality of life, genetic testing satisfaction, functional health, and medical outcomes.

Three themes were found across studies reporting PROs collected using general surveys (see Table 7): (1) self-regulation and decision making (e.g., utilization of risk-mitigating procedures such as prophylactic surgery); (2) experience and satisfaction with the genetic testing process; and (3) distress, knowledge, and risk perception.

Four themes were found across studies reporting qualitative PRO data (see Table 5): (1) feelings of satisfaction or readiness about future personal and family communication and planning; (2) genetic disease awareness and knowledge gain; (3) concern about insurance discrimination and stigmatization, as well as the cost of testing or care; (4) psychological and emotional feelings of intrusion, avoidance, shock, disbelief, distrust, curiosity, emotional coping, feeling emotional, psychosocial impact, relief, gratitude, regret, and fear.

PROs elements captured in this review that overlap with the personal utility elements described by Kohler et al. include coping, future planning and preparedness (family and personal), well-being and quality of life, knowledge gain, family communication, discrimination and stigma, and social support (see Table 8) [6].

The PRO themes and data collection methods identified in our review can inform engagement and consensus- or capacity-building initiatives that aim to (1) reconcile themes within the present literature review against the personal utility identified by Kohler et al. and possibly others; (2) build on the current literature to contextually define PROs following genetic testing for breast and ovarian cancer, Lynch syndrome, and FH; (3) determine if the themes identified are sufficient in substance or if more substance or exploration of themes are needed for further definition; and (4) channel these themes and definitions into real-world scenarios, data standards and collection methods, and other important practices or protocols moving forward.

Dobrozsi and Panepinto proposed a conceptual framework to incorporate PROs as measures that define patient symptoms and function with the goal of tailoring therapies, improving patient outcomes, improving patient–provider communication, and improving health care provider quality and performance [44]. By implementing this conceptual framework and describing and elucidating the role of PROs within the clinical context of genetic testing, it may become possible to understand the full value and personal utility of genetic testing for these conditions from the patient perspective. What is required in tandem, however, is more precision toward the adaptation or development of PROMs in this interventional context. As Victorson stated in a recent presentation outlining challenges to uses of PROs (e.g., poor validity, lack of sensitivity and specificity to specific interventions, low meaningfulness in certain contexts, and inappropriate survey/questionnaire delivery mechanisms), ‘PROs are not without their challenges… All of these things are important considerations to consider when creating a new PRO measure’ [45].

Indeed, PROs hold important implications for real-world clinical, research, market, reimbursement, and policy settings. PROs can inform and affect important and consequential areas of clinical practice and research, policy, pricing and reimbursement negotiations, and regulation concerning genetic testing for these conditions. For example, the PROs identified in this review can be cross-examined in current authoritative lists of validated PROMs, such as the (1) Patient-Reported Outcomes Measurement Information System, and (2) validated PROM list provided by the Australian Commission on Safety and Quality in Healthcare that covers high-burden cancers and cardiovascular diseases [46,47]. Additionally, PROs, being real-world evidence and clinical outcome assessment measures, informed the development of guidance documents focused on integrating patient experience data into drug and/or diagnostic test development processes (e.g., development processes for pharmacogenomic drugs and companion diagnostics). The drug development process mandated by the Prescription Drug User Fee Act VI (PDUFA VI) and Twenty-First Century Cures Act in the US is one such example that holds relevance [48]. Discussions have begun and continue today about how health authorities in France, the US, and the United Kingdom can integrate PROs, as a form of real-world evidence, into market authorization discussions and price and reimbursement negotiations [49,50].

In addition to analog surveys, interviews, and focus groups, PROs are and can be collected in both structured and unstructured data formats as digital measures. This can occur both within and outside of clinical settings using the internet or Internet of Things (IoT; e.g., data cloud hosts or servers), automated telephone systems, or downloadable applications such as mobile apps (digital PROs) [48,51,52]. There are benefits to collecting PROs digitally. For example, patients reporting digital PROs can report outcomes in real-time versus only during clinical appointments where there may be time and spatial and/or human resource challenges that might introduce confounding or other limitations into PRO data or collection methods.

There are risks, however, to the use and implementation of digital PROs following genetic testing, such as privacy and security risks in the event such PROs are collected and shared with third parties without patient awareness or consent. Privacy and security risks are especially concerning for patients reporting PROs following genetic testing, as such PROs can have implications for not just the patient but also their biological family members that may or may not be aware of the genetic condition being tested [53]. The sensitive and personal nature of PROs also renders PRO data vulnerable to re-identification if de-identified PRO data is shared with third parties. Novel approaches to collecting and analyzing structured and unstructured PRO data, such as the federated machine learning approach, can therefore be explored as potentially viable mechanisms for collecting digital PROs in a privacy-preserving manner following genetic testing [52].

There are three key limitations to our systematic literature review. First, our literature search was conducted in only three databases (PubMed/MEDLINE, EMBASE, and Yale University’s TRIP Medical Database). Future reviews could repeat our search methodology in other existing databases. Second, given that our review focused on a qualitative assessment of PROs following genetic testing for breast and ovarian cancer syndrome, Lynch syndrome, and FH, our methodological quality assessment of each study was from a qualitative standpoint. Future research could involve a methodological quality assessment of studies, particularly for studies reporting quantitatively measured PROs. Finally, our overall findings reflect an overrepresentation of or bias toward studies reporting (1) PROs following genetic testing for breast and ovarian cancer and studies conducted in the USA, and (2) PROs from a majority of participants of White/Caucasian/Northern European/Anglo-Saxon descent. Further research should examine PROs following genetic testing for Lynch syndrome and FH within and outside of the USA to enrich our review findings. Additionally, given that 11 of the 24 studies that met our inclusion criteria reported a majority of participants of White/Caucasian/Northern European/Anglo-Saxon descent, future studies should collect and assess the scope and range of PROs following genetic testing in more racially and ethnically diverse populations. Lastly, recent PRO studies have described how the coronavirus disease 2019 (COVID-19) pandemic has negatively impacted the daily quality of life, mental health, and medical management of some cancer patients [54,55]. Therefore, future work should also examine, through PRO assessments, the intersectional impact of COVID-19 and genetic testing on breast and ovarian cancer syndrome, Lynch syndrome, and FH patients.

## 5. Conclusions

PROs are important data endpoints that can reflect or convey patient experiences, feelings, thoughts, and journeys across time and health settings. PRO data collection methods are, therefore, important and necessary to understand or perhaps even predict patient health beliefs, risks, and behaviors following genetic testing. This review presents the state of evidence regarding PRO data collection methods and themes following genetic testing for FH, breast and ovarian cancer, and Lynch syndrome and offers recommendations for future clinical and policy engagement and research. Clinicians, patients, regulators, policymakers, and other stakeholders are therefore encouraged to disseminate and implement these findings across key clinical, educational, and policy settings and discussions.

## Figures and Tables

**Table 1:** Main characteristics of patient-reported outcomes (PROs outlined by Cella et al.) [5].

PRO Category	Main Characteristics	Main Strengths	Main Limitations
Health-related quality of life (HRQL)	- Is multidimensional - Can be generic or condition-specific	- Yields a global summary of well-being	- May not be considered a sufficiently specific construct
Functional status	- Reflects ability to perform specific activities	- Can be used in addition to performance-based measures of function	- May reflect variations in self-reported capability and actual performance of activities
Symptoms and symptom burden	- Are specific to type of symptom of interest - May identify symptoms not otherwise captured by medical workup	- Are best assessed through self-report	- May fail to capture general, global aspects of well-being considered important to patients
Health behaviors	- Are specific to type of behavior - Typically measure frequency of behavior	- Target specific behavior categories	- Validity may be affected by social desirability - May produce potential patient discomfort in reporting socially undesirable behaviors
Patient Experience	- Concerns satisfaction with health care delivery, treatment recommendations, and medications (or other therapies) - Reflects actual experiences with health care services- Fosters patient activation	- Is an essential component of patient-centered care - Is valued by patients, families, and policymakers - Relates to treatment adherence - Relates to health behaviors and health outcomes	- May be a complex, multidimensional construct - Requires confidentiality to ensure patient comfort in disclosing negative experiences - Does not provide sufficient evidence that activation enhances health care decision making

**Table 2:** Summary of study characteristics.

Study ID	Country	Genetic Disease Focus	Qualitative/ Quantitative/ Mixed Methods
Beri et al. (2019) [21]	USA	Breast and ovarian cancer	Quantitative
Bradbury et al. (2020) [22]	USA	Breast and ovarian cancer	Quantitative
Croyle et al. (1997) [20]	USA	Breast and ovarian cancer	Quantitative
deWit et al. (1998) [18]	Netherlands	Breast and ovarian cancer	Quantitative
Esplen et al. (2015) [23]	Canada	Lynch syndrome	Quantitative
Hallowell et al. (2004) [24]	UK	Breast and ovarian cancer	Qualitative
Hughes Halbert et al. (2011) [25]	USA	Breast and ovarian cancer	Quantitative
Jones et al. (2018) [16]	USA	Familial hypercholesterolemia	Qualitative
Lee et al. (2002) [26]	USA	Breast and ovarian cancer	Not Reported
Lerman et al. (1996) [27]	USA	Breast and ovarian cancer	Mixed methods
Lerman et al. (1998) [28]	Canada/USA	Breast and ovarian cancer	Mixed methods
Luba et al. (2018) [29]	USA	Lynch syndrome	Quantitative
MacLeod et al. (2014) [30]	UK	Breast and ovarian cancer	Qualitative
Mallen et al. (2021) [31]	USA	Breast and ovarian cancer	Qualitative
Manchanda et al. (2019) [32]	UK	Breast and ovarian cancer	Quantitative
Meiser et al. (2018) [33]	Australia	Breast and ovarian cancer	Quantitative
Mella et al. (2017) [34]	Italy	Breast and ovarian cancer	Quantitative
Palmquist et al. (2010) [35]	USA	Lynch Syndrome	Qualitative
Pang et al. (2018) [17]	Australia	Familial hypercholesterolemia	Not reported
Schneider et al. (2018) [36]	USA	Lynch syndrome	Qualitative
Tiller et al. (2020) [7]	Australia	Lynch syndrome Breast and ovarian cancer	Mixed methods
Underhill-Blazey et al. (2020) [37]	USA	Breast, ovarian, and endometrial/uterine cancer	Mixed methods
Voorwinden et al. (2012) [38]	Netherlands	Lynch syndromeBreast and ovarian cancer	Quantitative
Werner-Lin et al. (2012) [39]	USA	Breast and ovarian cancer	Qualitative

**Table 3:** Studies reporting PROs from validated PROM instruments following genetic testing for breast and ovarian cancer syndrome or Lynch syndrome.

Study ID	Genetic Disease	PROM Instrument Used
Beri et al. (2019) [21]	Breast and ovarian cancer	- Cancer Genetics Knowledge Scale - State-Trait Anxiety Inventory - Hospital Anxiety and Depression Scale - Impact of Event Scale - Multidimensional Impact of Cancer Risk Assessment
Croyle et al. (1997)	Breast and ovarian cancer	- State-Trait Anxiety Inventory - Impact of Event Scale
deWit et al. (1998) [18]	Breast and ovarian cancer	- Impact of Event Scale
Esplen et al. (2015) [23]	Lynch syndrome	- Center for Epidemiologic Studies for Depression Scale - State-Trait Anxiety Inventory - Impact of Event Scale - Perception of Lifetime Risk for Colorectal Cancer - Colonoscopy Screening - Ways of Coping Questionnaire - Social Support Questionnaire - Quality of Life Index - Demographic and Medical Information Questionnaire
Hughes Halbert et al. (2011) [25]	Breast and ovarian cancer	- Multidimensional Impact of Cancer Risk Assessment
Lerman et al. (1996) [27]	Breast and ovarian cancer	- Center for Epidemiologic Studies for Depression Scale - Medical Outcomes Study
Lerman et al. (1998) [28]	Breast and ovarian cancer	- Center for Epidemiologic Studies for Depression Scale - Intrusion Subscale of the Revised Impact of Event Scale
Manchanda et al. (2019) [32]	Breast and ovarian cancer	- Hospital Anxiety and Depression Scale - Health Anxiety Inventory - Multidimensional Impact of Cancer Risk Assessment - SF12 questionnaire (Physical Health Component Scale and Mental Health Component Scale)
Meiser et al. (2018) [33]	Breast and ovarian cancer	- Impact of Event Scale - Hospital Anxiety and Depression Scale - Decision Regret Scale (genetic testing choice and surgery choice)
Mella et al. (2017) [34]	Breast and ovarian cancer	- Hospital Anxiety and Depression Scale - Profile of Mood States - Emotional Thermometers (ad hoc instrument)
Underhill-Blazey et al. (2020) [37]	Breast, ovarian, and endometrial/uterine cancer	- Multidimensional Impact of Cancer Risk Assessment
Voorwinden et al. (2012) [38]	Lynch syndrome Breast and ovarian cancer	- General Health Questionnaire-12 - Cancer Worry Scale

**Table 4:** Studies using validated general surveys to collect and assess PROs following genetic testing for breast and ovarian cancer syndrome or Lynch syndrome.

Study ID	Genetic Disease	Survey Measures
Beri et al. (2019) [21]	Breast and ovarian cancer	- Perceptions of genetic counseling and testing experience (Patrick-Miller et al. 2013 and Pieterse et al. 2007) [40,41] - Intent to utilize services (mammography, breast MRI, colonoscopy, prophylactic surgeries [e.g. mastectomy and oophorectomy])
Bradbury et al. (2020) [22]	Breast and ovarian cancer	- Self-Regulation Theory of Health Behavior
Lerman et al. (1996) [27]	Breast and ovarian cancer	- Impact of receipt of BRCA1 test results on decisions about prophylactic mastectomy and prophylactic oophorectomy
Luba et al. (2018) [29]	Lynch syndrome	- Satisfaction
Meiser et al. (2018) [33]	Breast and ovarian cancer	- Test-Related Distress - Positive Experiences - Uptake of bilateral mastectomy or risk-reducing bilateral salpingo-oophorectomy
Underhill-Blazey et al. (2020)	Breast, ovarian, and endometrial/uterine cancer	- Genetic Counseling and Testing Satisfaction (Bradbury et al., 2016) [42] - KnowGene (Underhill-Blazey et al., 2019) [43]
Voorwinden et al. (2012) [38]	Lynch syndrome; Breast and ovarian cancer	- Knowledge - Risk perception - Decision-making

**Table 5:** Studies reporting qualitative data or other unstructured PRO data following genetic testing for breast and ovarian cancer syndrome, Lynch syndrome, and/or familial hypercholesterolemia (FH).

Study ID	Genetic Disease	Data Collection Method	PROs Reported
Hallowell et al. (2004) [24]	Breast and ovarian cancer	Qualitative interviews	- Reflecting on the past: the impact of a cancer diagnosis on self-identity - Looking to the future: genetic risk and identity - Accounting for the past and predicting the future: women’s motivations for undergoing genetic testing - Intrusion - Avoidance
Jones et al. (2018) [16]	FH	Qualitative interviews	- Understanding of FH (i.e., the inherited nature of FH) - Concerns about increased risk for heart attacks - Confusion and uncertainty for future medical care - Importance of genetic test results for family members. - Communicating with family members to understand family history of heart disease. - Feelings of shock from incidental genetic test findings (i.e., non-paternity)
Lee et al. (2002) [26]	Breast and ovarian cancer	Not reported	- Concern about insurance discrimination - Cost (free/self-pay/insurance)
Lerman et al. (1996) [27]	Breast and ovarian cancer	Telephone interview assessment	- Knowledge of genetic disease - Disbelief in cancer prevention - Worry about losing insurance coverage - Concern about accuracy of test results - Distrust in trust modern medicine - Curiosity about need for increased screening - Planning for the future for reassurance (i.e., making surgery and/or childbearing decisions, understanding children’s risk) - Poor emotional coping
MacLeod et al. (2014) [30]	Breast and ovarian cancer	Qualitative interviews	- Expecting to be gene-positive; preparing for possibility of bad news - Not a difficult decision; relieve uncertainty - Time for action; alter course of disease - Parental attitudes to testing; parental best interests at heart - Initial shock
Mallen et al. (2021)	Breast and ovarian cancer	Qualitative interviews	- Knowledge of genetic testing availability to learn disease risk - Beliefs and attitudes; inquiry and advocacy - Insurance coverage and out-of-pocket cost - Knowledge about genetic risks to self or family - Emotional reactions to the idea of learning about genetic risk may deter the pursuit of genetic testing - Positive appraisal; focusing on something favorable about their situation - Results not shared widely; shared only with a small immediate circle of close friends and family - Family impact of test results
Palmquist et al. (2010) [35]	Lynch Syndrome	Qualitative interviews	- Understanding the link between risk perception and cancer prevention - Understanding disease risk - Family history and cancer experiences in the formation of risk perception - Availability and accessibility to genetic testing
Pang et al. (2018) [17]	FH	Not reported	- Concern about the stigmatization of genetic testing - Reasons why parents did not provide consent for genetic testing: (i) wished children to make their own decision regarding testing after age 18 years; (ii) both parents could not reach unanimous decision
Schneider et al. (2018) [36]	Lynch syndrome	Qualitative interviews	- Facilitators and barriers to care coordination and receipt - Familiarity with Lynch syndrome and engagement with screening recommendations - Approach to and support with surveillance recommendations - Informing and communicating with family members - Appreciation for being able to inform family members - Relief - Gratitude - Regret - Fear - Feeling emotional
Tiller et al. (2020) [7]	Lynch syndrome; breast and ovarian cancer	Qualitative interviews	- Difficulty accessing insurance
Underhill-Blazey et al. (2020) [37]	Breast, ovarian, and endometrial/uterine cancer	Focus group interviews	- Genetic knowledge - Understanding complex genetic testing results - Communicate novel developments and recommendations to patients who receive a variant of unknown significance test result - Satisfaction - Preparing to communicate genetic test results to family members- Facilitating cascade testing (when necessary) - Psychosocial impact
Werner-Lin et al. (2012) [39]	Breast and ovarian cancer	Qualitative interviews	- Still learning - Feeling vulnerable to an impending cancer diagnosis and pressure to act before crossing a threshold into territory perceived as unsafe - Making active lifestyle choices to support healthy living since learning their mutation status - Navigating to and through genetic counseling and/or testing and risk management decision making - Actively seeking readily accessible resources to clarify and facilitate risk management

**Table 6:** Quality assessment of studies reporting PROs following genetic testing for breast and ovarian cancer syndrome, Lynch syndrome, and/or familial hypercholesterolemia.

Study ID	Was There a Clear Statement of the Aims of the Research?	Is a Qualitative Methodology Appropriate?	Was the Research Design Appropriate to Address the Aims of The Research?	Was the Recruitment Strategy Appropriate to the Aims of The Research?	Were the Data Collected in a Way That Addressed the Research Issue?	Has the Relationship between Researcher and Participants Been Adequately Considered?	Have Ethical Issues Been Taken into Consideration?	Was the Data Analysis Sufficiently Rigorous?	Is There a Clear Statement of Findings?	How Valuable Is the Research?
Beri et al. (2019) [21]	Yes	No	Yes	Yes	Yes	Yes	Yes	Yes	Yes	Major
Bradbury et al. (2020) [22]	Yes	No	Yes	Yes	Yes	Yes	Yes	Yes	Yes	Major
Croyle et al. (1997)	Yes	No	Yes	Yes	Yes	Cannot Tell	Yes	Yes	Yes	Major
deWit et al. (1998) [18]	Yes	No	Yes	Yes	Yes	Yes	Yes	Yes	Yes	Major
Esplen et al. (2015) [23]	Yes	No	Yes	Yes	Yes	Yes	Yes	Yes	Yes	Major
Hallowell et al. (2004) [24]	Yes	Yes	Yes	Yes	Yes	Yes	Yes	Yes	Yes	Major
Hughes Halbert et al. (2011) [25]	Yes	No	Yes	Yes	Yes	Yes	Yes	Yes	Yes	Major
Jones et al. (2018) [16]	Yes	Yes	Cannot tell	Yes	Cannot tell	Yes	Yes	Yes	Yes	Major
Lee et al. (2002) [26]	Yes	Cannot tell	Cannot tell	Yes	Cannot tell	Yes	Yes	Cannot tell	Yes	Minor
Lerman et al. (1996) [27]	Yes	No	Yes	Yes	Yes	Yes	Yes	Yes	Yes	Major
Lerman et al. (1998) [28]	Yes	No	Yes	Yes	Yes	Yes	Yes	Yes	Yes	Major
Luba et al. (2018) [29]	Yes	No	Yes	Yes	Yes	Yes	Yes	Yes	Yes	Major
MacLeod et al. (2014) [30]	Yes	Yes	Yes	Yes	Yes	Yes	Yes	Yes	Yes	Major
Mallen et al. (2021)	Yes	Yes	Yes	Yes	Yes	Yes	Yes	Yes	Yes	Major
Manchanda et al. (2019) [32]	Yes	No	Yes	Yes	Yes	Yes	Yes	Yes	Yes	Major
Meiser et al. (2018) [33]	Yes	No	Yes	Yes	Yes	Yes	Cannot tell	Yes	Yes	Major
Mella et al. (2017) [34]	Yes	No	Yes	Yes	Yes	Yes	Yes	Yes	Yes	Major
Palmquist et al. (2010) [35]	Yes	Yes	Yes	Yes	Yes	Yes	Yes	Yes	Yes	Major
Pang et al. (2018) [17]	Yes	Cannot tell	Yes	Yes	Yes	Yes	Yes	Yes	Yes	Minor
Schneider et al. (2018) [36]	Yes	Yes	Yes	Yes	Yes	Yes	Yes	Yes	Yes	Major
Tiller et al. (2020) [7]	Yes	No	Yes	Yes	Yes	Yes	Yes	Yes	Yes	Major
Underhill-Blazey et al. (2020) [37]	Yes	Yes	Yes	Yes	Yes	Yes	Yes	Yes	Yes	Major
Voorwinden et al. (2012) [38]	Yes	No	Yes	Yes	Yes	Yes	Yes	Yes	Yes	Major
Werner-Lin et al. (2012) [39]	Yes	Yes	Yes	Yes	Yes	Yes	Yes	Yes	Yes	Major

**Table 7:** Studies using general surveys to collect and assess PROs following genetic testing for breast and ovarian cancer syndrome or Lynch syndrome.

Study ID	Genetic Disease	Survey Measures
Beri et al. (2019) [21]	Breast and ovarian cancer	- Perceptions of genetic counseling and testing experience (Patrick-Miller et al. 2013 and Pieterse et al. 2007) [40,41] - Intent to utilize services (mammography, breast MRI, colonoscopy, prophylactic surgeries (e.g., mastectomy and oophorectomy))
Bradbury et al. (2020) [22]	Breast and ovarian cancer	- Self-Regulation Theory of Health Behavior
Lerman et al. (1996) [27]	Breast and ovarian cancer	- Impact of receipt of BRCA1 test results on decisions about prophylactic mastectomy and prophylactic oophorectomy
Luba et al. (2018) [29]	Lynch syndrome	- Satisfaction
Meiser et al. (2018) [33]	Breast and ovarian cancer	- Test-related distress - Positive experiences - Uptake of bilateral mastectomy or risk-reducing bilateral salpingo-oophorectomy
Underhill-Blazey et al. (2020) [37]	Breast, ovarian, and endometrial/uterine cancer	- Genetic counseling and testing satisfaction (Bradbury et al., 2016) [42] - KnowGene (Underhill-Blazey et al., 2019) [43]
Voorwinden et al. (2012) [38]	Lynch syndrome; breast and ovarian cancer	- Knowledge - Risk perception - Decision-making

**Table 8:** Summary, comparison, and alignment of the current state of PRO collection methods and themes * following genetic testing for breast and ovarian cancer syndrome, Lynch syndrome, and/or familial hypercholesterolemia and Kohler et al. elements of personal utility.

Validated Patient-Reported Outcome Measure Surveys	General Surveys	Qualitative Interviews and Focus Groups	Kohler et al.
Psychological, mood, emotional function or state (i.e., feelings of anxiety, depression, distress, regret, and worry)		Psychological and emotional feeling of intrusion, avoidance, shock, disbelief, distrust, curiosity, emotional coping, feeling emotional, psychosocial impact, relief, gratitude, regret, and fear	
Knowledge and perceptions of their lifetime or genetic disease risk, uncertainty about managing their genetic disease risk, and health screening behaviors	Self-regulation and decision making (e.g., utilization of risk-mitigating procedures such as prophylactic surgery) Distress, knowledge, and risk perception	Genetic disease awareness and knowledge gain	Mental preparation Feelings of responsibility Value of information Knowledge of condition Self-knowledge Curiosity
Coping style or mechanisms, social functioning and support, quality of life, genetic testing satisfaction, functional health, and medical outcomes	Experience and satisfaction with the genetic testing process	Feelings of satisfaction or readiness about future personal and family communication and planning	To enhance coping Improved spiritual well-being Ability for future planning Reproductive autonomy Communication with relatives Change in social support
		Concern about insurance discrimination and stigmatization Concern about cost of testing or care	Concern about discrimination and stigma Concern about privacy
			Research altruism

* Grey-colored fields in the table represent either a lack of PRO content alignment or overall evidence gaps.

## Data Availability

Not applicable.
